# Insulin-like peptides play distinct roles in nutrient-dependent plasticity in *Drosophila*

**DOI:** 10.1093/g3journal/jkaf301

**Published:** 2025-12-12

**Authors:** Michelle A Henstridge, Bowen Slater, Jade R Kannangara, Christen K Mirth

**Affiliations:** School of Biological Sciences, Monash University, Clayton, Victoria 3800, Australia; School of Biological Sciences, Monash University, Clayton, Victoria 3800, Australia; School of Biological Sciences, Monash University, Clayton, Victoria 3800, Australia; School of Biological Sciences, Monash University, Clayton, Victoria 3800, Australia

**Keywords:** insulin-like peptides, nutrition, body size, development time, *Drosophila melanogaster*

## Abstract

The highly conserved insulin signaling pathway regulates growth and development time in response to nutrition across metazoans. The fruit fly, *Drosophila melanogaster,* has 7 insulin-like peptides, which bind to a single insulin receptor and are differentially expressed across development time, organs, and with nutritional conditions. However, whether individual insulin-like peptides play specific roles in controlling growth remains unknown. Recent studies have revealed that, in addition to the caloric content of the diet, the ratio of protein to carbohydrates in the diet plays a key role in regulating life history traits. Furthermore, individual insulin-like peptides vary in their expression profiles according to nutrient conditions. Whether these differences in expression have any functional significance to animal life history traits remains unclear. Here, we report that reducing the protein content of the larval diet through macronutrient restriction—where the calories lost from protein dilution are offset by increased carbohydrate content—results in a more pronounced developmental delay compared to caloric restriction—where both protein and carbohydrate concentrations are reduced. We further reveal that these two diet types result in notable differences in the expression levels of *Drosophila insulin-like peptides 2*, *3*, and *5*, and observe distinct phenotypic responses of individual *insulin-like peptide* mutants raised on each diet type. Taken together, our findings highlight the distinct roles of individual insulin-like peptides in regulating growth and development time in response to changes in dietary macronutrients, and provide key insights into the molecular mechanisms controlling nutritional plasticity in *Drosophila*.

## Introduction

The regulation of growth and development time in response to nutrition is mediated by the insulin/IGF-signaling (IIS) pathway, a highly conserved receptor–tyrosine kinase pathway found across all metazoans (Pertseva and Shpakov 2002). Under high nutrition, insulin is secreted into circulation and binds to receptors on peripheral cells, initiating a signal transduction cascade resulting in the activation or repression of different genes that control growth (for review see Suzawa and Bland 2023). Interestingly, many species are known to have multiple insulin-like peptides present in their genome (Pierce et al. 2001; Riehle et al. 2006; Gronke et al. 2010) and recent evidence suggests that these peptides may differ in both their expression patterns and biological functions (Semaniuk et al. 2020).

In *Drosophila,* 7 insulin-like peptides (dILPs 1–7) and a relaxin-like peptide (dILP8) have been identified (Brogiolo et al. 2001; Gronke et al. 2010; Garelli et al. 2012; Colombani et al. 2015). dILPs 1–7 bind to a single insulin receptor in this species (InR; Brogiolo et al. 2001; Garofalo 2002). The various *dilp* genes are expressed in different larval and adult tissues and cell types (Brogiolo et al. 2001; Ikeya et al. 2002; Semaniuk et al. 2020), and therefore are likely to have different functions. For example, dILP6 is produced in the larval fat body and regulates growth during starvation and nonfeeding states (Okamoto et al. 2009; Slaidina et al. 2009; Bai et al. 2012). The dILPs thought to regulate most of the nutritional responses, dILPs 2, 3, and 5, are secreted by the insulin-producing cells (IPCs) within the brain (Brogiolo et al. 2001; Rulifson et al. 2002; Geminard et al. 2009). Even though they are produced in the same cells, distinct classes of nutrients regulate dILP2 and dILP3. While dILP2 is secreted in response to amino acids (Geminard et al. 2009), dILP3 secretion is stimulated by sugars (Kim and Neufeld 2015). Despite numerous studies on the roles of dILPs 2, 3, and 5, it is still not clear how these three peptides differ in their expression levels and function in response to changes in nutrition.

For many years research has focused on how total food intake influences development time and body size, rather than the specific composition of the food. Thus, diet manipulations have most often diluted all components of the diet, known as caloric restriction. In recent years, our understanding of the role of macronutrients in shaping the fitness of an organism has been greatly advanced by the development of nutritional geometry, a multidimensional modeling approach which enables the complex interactions between macronutrients, such as protein and carbohydrates, and life history traits to be examined (Lee et al. 2008; Simpson and Raubenheimer 2012). Studies using nutritional geometry in *Drosophila* have revealed that traits like metabolism (Alton et al. 2020), development time (Rodrigues et al. 2015; Kutz et al. 2019; Chakraborty et al. 2020; Kim et al. 2020; Min et al. 2021), body size (Rodrigues et al. 2015; Kutz et al. 2019; Chakraborty et al. 2020; Kim et al. 2020; Min et al. 2021), fecundity (Lee et al. 2008; Kim et al. 2020; Min et al. 2021), and lifespan (Lee et al. 2008; Skorupa et al. 2008; Kim et al. 2020; Min et al. 2021) are regulated more by specific ratios of protein to carbohydrates in the diet rather than total caloric content.

While the mechanism through which the protein to carbohydrate ratio of the diet affects these life history traits remains unknown, there are several lines of evidence to suggest that it involves changes in *dilp* expression (Kim and Neufeld 2015; Post and Tatar 2016; McDonald et al. 2021). In a recent study, McDonald et al. (2021) used a nutritional geometry framework to explore the effects of dietary protein and carbohydrate content on body size and the expression of genes that are transcriptionally regulated by the IIS pathway, including *dilps 2*, *3*, *5*, and *8*. This research revealed dramatic differences in the expression patterns of each *dilp* across the nutritional landscape in larvae, revealing a complex relationship between sex, diet, and *dilp* expression (McDonald et al. 2021).

Here, we aimed to test whether the three brain-derived dILPs (dILPs 2, 3, and 5) are functionally different from one another by exploring whether they mediate different responses to diet in larval and adult life history traits. To do this, we compared the effects of macronutrient restriction, in which the calories lost from protein dilution are substituted by increasing the amount of carbohydrates, to caloric restriction, in which both protein and carbohydrate are diluted, on survival, development time, and body size. We then examined how larval expression levels of *dilps 2*, *3*, and *5* changed across protein concentrations and between diet types. Finally, we assessed how mutations in either *dilp 2*, *3*, or *5* altered the phenotypic response to diet. Understanding the precise role of each dILP is critical to further our understanding of the mechanisms controlling nutrient-dependent plasticity in *Drosophila* and other organisms.

## Materials and methods

### *Drosophila* stocks and maintenance

The following stocks were used: *w^1118^* (RRID:BDSC_5905), *w^1118^;TI{TI}ilp2^1^* (RRID:BDSC_30881), *w^1118^;TI{TI}ilp3^1^* (RRID:BDSC_30882), and *w^1118^;TI{TI}ilp5^1^* (RRID:BDSC_30884). FlyBase was used as an essential reference tool throughout this study (Gramates et al. 2022; Ozturk-Colak et al. 2024). All stocks were maintained at 25 °C on a sucrose–yeast media containing, per liter: 100 g yeast (MP biomedicals, Cat. No. 903312), 50 g sucrose (Bundaberg), 10 g agar (Gelita Australia), 30 mL Nipagen (10% in ethanol), and 3 mL propionic acid (Merck).

### Experimental diets

Experimental diets were based on manipulating the ingredients in a standard sucrose–yeast diet (Table 1; see recipe above), which contains a protein to carbohydrate (P:C) ratio of 1:2 and a caloric concentration of 495 calories/L. This control diet (1:2 or 100%) was then altered in two different ways: (i) caloric restriction, which reduced the caloric content of the diet to 50 and 25% of control food by reducing the amount of both sucrose and yeast while maintaining a constant P:C ratio (Table 1), or (ii) macronutrient restriction, in which the calories from yeast were substituted by increasing the amount of sucrose, altering the P:C ratio to 1:5 and 1:10 while maintaining a constant number of calories (Table 1).

**Table 1.** Composition of experimental diets.

	Amount (g/L)	Protein (g/L)	Carbs (g/L)	P + C	Calories
*Control food (1:2/100%)*					
Agar	10	0	0		
Sucrose	50	0	50		
Yeast	100	45	33		
Total		45	83	128	495
*Caloric restriction (50%)*					
Agar	10	0	0		
Sucrose	25	0	25		
Yeast	50	22.5	16.5		
Total		22.5	41.5	64	247.5
*Caloric restriction (25%)*					
Agar	10	0	0		
Sucrose	12.5	0	12.5		
Yeast	25	11.25	8.25		
Total		11.25	20.75	32	123.75
*Macronutrient restriction (1:5)*					
Agar	10	0	0		
Sucrose	89	0	89		
Yeast	50	22.5	16.5		
Total		22.5	105.5	128	495
*Macronutrient restriction (1:10)*					
Agar	10	0	0		
Sucrose	108.5	0	108.5		
Yeast	25	11.25	8.25		
Total		11.25	116.75	128	495

### Survival, development time, and body size analysis

Twenty-four hours after a 4 h lay on apple juice agar (50 g Agar [Gelita Australia], 600 mL apple juice [Golden Circle], and 30 mL Nipagen [10% in ethanol] per liter) supplemented with yeast paste, first-instar larvae were placed into vials containing fly media (see recipe above) and scored every 8 h for the time taken to reach pupariation. At least 8–10 vials each containing 30 individual larvae for experiments using *w^1118^* or 10 larvae for *dilp* mutant/heterozygote experiments were collected for each genotype and diet.

**Fig. 1.**
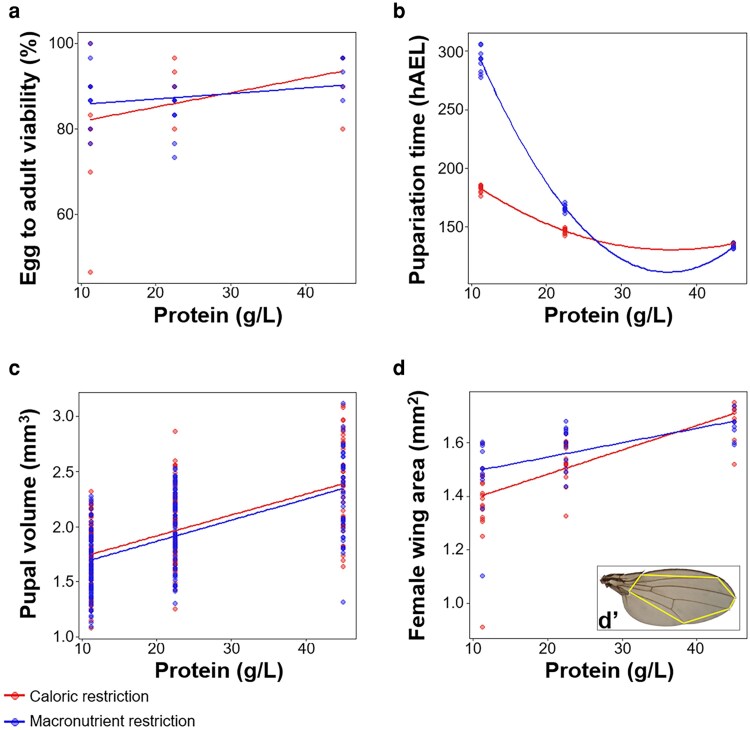
Dietary composition effects egg to adult viability, development time, and body size. a) Egg to adult viability increases with increasing protein concentration. No differences in egg to adult viability were observed between larvae raised on the caloric restriction diet (red line) and the macronutrient restriction diet (blue line). *n* = 5–10 groups of 30 animals for all diets. b) Pupariation time decreases with increasing protein concentration, with a more profound effect in larvae raised on the macronutrient restriction diet. hAEL = hours after egg lay. *n* = 5–10 groups of at least 26 individuals for all diets. c, d) Final body size, measured by pupal volume (c) and female wing area (d), increases with increasing protein concentration. Subtle differences in wing area were observed between the 2 diet types. (d’) Representative wing image showing the landmarks (gray circles) used to calculate wing area. *n* = 4 groups of 2 to 9 individuals for all diets. Alpha blending was applied to all graphs to indicate overlapping data points; darker areas represent higher point density.

For measurements of pupal volume, digital images of individual pupae were captured 7 days after pupariation using a Leica M165 FC stereoscope at 3.2× magnification and a Leica DFC450 C digital camera. All pupal images were analyzed using ImageJ software, and pupal volume was calculated assuming pupae are ellipsoid shaped using the formula, *V* = 3/4 × π × (L/2) × (W/2)^2^, where L is pupal length and W is pupal width, as described previously (Ghosh et al. 2022). When measuring wing area, adult female flies were collected following eclosion and preserved in SH solution (70% ethanol and 30% glycerol) after their wings had fully expanded and hardened. The left wing was dissected off each body in SH solution under a Leica MZZ5 microscope, mounted onto microscope slides and digital images of the wings were captured at 5× magnification using a Leica M165 FC stereoscope and a Leica DFC450 C digital camera. All wing images were analyzed using ImageJ software, and wing area was calculated using landmarks from vein intercepts (Fig. 1d’) as per Pocas et al. (2022).

### Insulin-like peptide gene transcript quantification

To obtain newly molted third instar larvae for quantification of *dilp* expression levels, eggs were collected over a 4 h laying interval on 55 mm diameter petri dishes containing each diet. Approximately 72–96 h after egg lay (depending on diet type), larvae were carefully staged within 2 h of the molt to the third larval instar as per Chakraborty et al. (2021). For each biological replicate, 10 to 15 third instar larvae (anterior end only) were snap frozen before RNA was extracted and DNAse treated using Direct-zol RNA MiniPrep kit (Zymo Research). Complementary DNA was synthesized using Tetro reverse transcriptase (Bioline) by priming 5 µg of RNA with oligo-dT and random hexamers. Quantitative PCRs were performed in duplicate on a QuantStudio 3 (ThermoFisher) using SensiMix Sybr (Bioline) and primers specific for *dilp2* (F—5′-ACG AGG TGC TGA GTA TGG TGT GCG-3′, R—5′-CAC TTC GCA GCG GTT CCG ATA TCG-3′), *dilp3* (F—5′-GTC CAG GCC ACC AAT GAA GTT G-3′, R—5′-CTT TCC AGC AGG GAA CGG TCT TCG -3′), *dilp5* (F—5′-TGT TCG CCA AAC GAG GCA CCT TGG-3′, R—5′-CAC GAT TTG CGG CAA CAG GAG TCG-3′), and *Rp49* (F—5′-GCC GCT TCA AGG GAC AGT ATC T-3′, R—5′-AAA CGC GGT TCT GCA TGA G-3′). Fold changes relative to *Rp49* were determined using the delta CT method, and means and standard errors were calculated from 3 to 5 biological replicates per genotype.

### Statistical analysis

Data was visualized and analyzed in R (Rstudio version 1.4.1103). To visualize the data, we used the Tidyverse package (Wickham et al. 2019). For egg to adult viability, we fit the data with generalized mixed effects models using a binomial distribution (lme4 package, Bates et al. 2015) and using egg to adult viability as a response variable. Development time and body size measurements were fitted with linear mixed effect models (lme4 package, Bates et al. 2015) using either pupariation time or pupal volume/wing area as response variables. Fixed effects included a second-order polynomial for protein concentration, in addition to diet type and genotype (where appropriate). Replicates were included as a random effect. Posthoc tests of the means and trends were conducted using the emmeans package (Lenth 2025). To quantify the amount of variation in the data explained by our models, we estimated both conditional ($R_{c}^{2}$, takes both fixed and random effects in the model into account) and marginal ($R_{m}^{2}$ takes only fixed effects in the model into account) R^2^ values using the performance package (Nakagawa and Schielzeth 2013).

## Results

### Dietary composition effects egg to adult viability, development time, and body size

To investigate how dietary composition effects larval viability, development time, and adult body size, we diluted the protein content of the larval diet in 2 ways: (i) caloric restriction, which reduces the caloric content of the diet to 50 and 25% of control food while maintaining a constant P:C ratio, or (ii) macronutrient restriction, in which the calories from protein are substituted by increasing the amount of carbohydrates. This alters the P:C ratio from 1:2 in the control food to 1:5 and 1:10 while maintaining a constant caloric content. We then measured how raising larvae of a control genotype (*w^1118^*) on these different diets effected viability, development time, and body size.

On both the caloric and macronutrient restriction diets, egg to adult viability increased with increasing protein concentration (Fig. 1a, Table 2); however, no significant differences in egg to adult viability were found between the two diet types (Fig. 1a, Table 2). Development time was found to significantly decrease with increasing protein concentration on both the caloric and macronutrient restriction diets (Fig. 1b, Table 2). Interestingly, we observed a more severe developmental delay when larvae were raised on the low-protein macronutrient-restriction diets compared to the low-protein caloric-restriction diets (Fig. 1b, Table 2).

**Table 2.** The effect of dietary manipulations on survival, pupariation time, and body size.

	Df	Viability	Pupariation time	Pupal volume	Wing area
		χ^2^ value	χ^2^ value	χ^2^ value	χ^2^ value
Protein	1	10.5045**	91.525***	209.707***	2.574 × 10^−13^***
Diet	1	0.5874	42.177***	2.2132	0.0411*
Protein:diet	1	2.1628	27.104***	0.0073	0.0652

Significance codes: *P < 0.05, **P < 0.01, ***P < 0.001.

Viability $R_{c}^{2}$ = 0.572, $R_{m}^{2}$ = 0.409; pupariation time $R_{c}^{2}$ = 0.952, $R_{m}^{2}$ = 0.951; pupal volume $R_{m}^{2}$ = 0.348; wing area $R_{c}^{2}$ = 0.614, $R_{m}^{2}$ = 0.589.

We found that final body size, as measured by pupal volume, increased with increasing protein concentration on both the caloric and macronutrient restriction diets (Fig. 1c, Table 2). However, despite the different effect diet type had on development time, we did not observe a significant difference on the impact of protein concentration on pupal volume between caloric and macronutrient restriction diets (Fig. 1c, Table 2). Like pupal volume, female wing area also increased with increasing protein concentration on both the caloric and macronutrient restriction diets (Fig. 1d, Table 2). However, subtle, but significant differences in female wing area were observed between the 2 diet types (Fig. 1d, Table 2). These differences were most evident at the lowest protein concentrations. Together, these data suggest that diluting the protein content of the larval diet via macronutrient restriction has a different effect on larval growth and development time compared to protein dilution via caloric restriction.

### *dilp* expression patterns differ in response to dietary protein concentration and diet type

Previous studies have revealed that dILPs secreted by the insulin-producing cells differ in the way in which they respond to changes in dietary nutrient concentration (Kim and Neufeld 2015; Post and Tatar 2016; McDonald et al. 2021). Thus, the differences in development time we observed when *w^1118^* larvae were raised on the caloric and macronutrient restriction diets led us to hypothesize that these two methods of protein dilution may alter insulin signaling and, more specifically, *dilp* expression in different ways. To explore this idea, we measured *dilp2*, *3*, and *5* mRNA levels in third-instar *w^1118^* larvae that had been raised on either the caloric or macronutrient restriction diets.

**Fig. 2.**
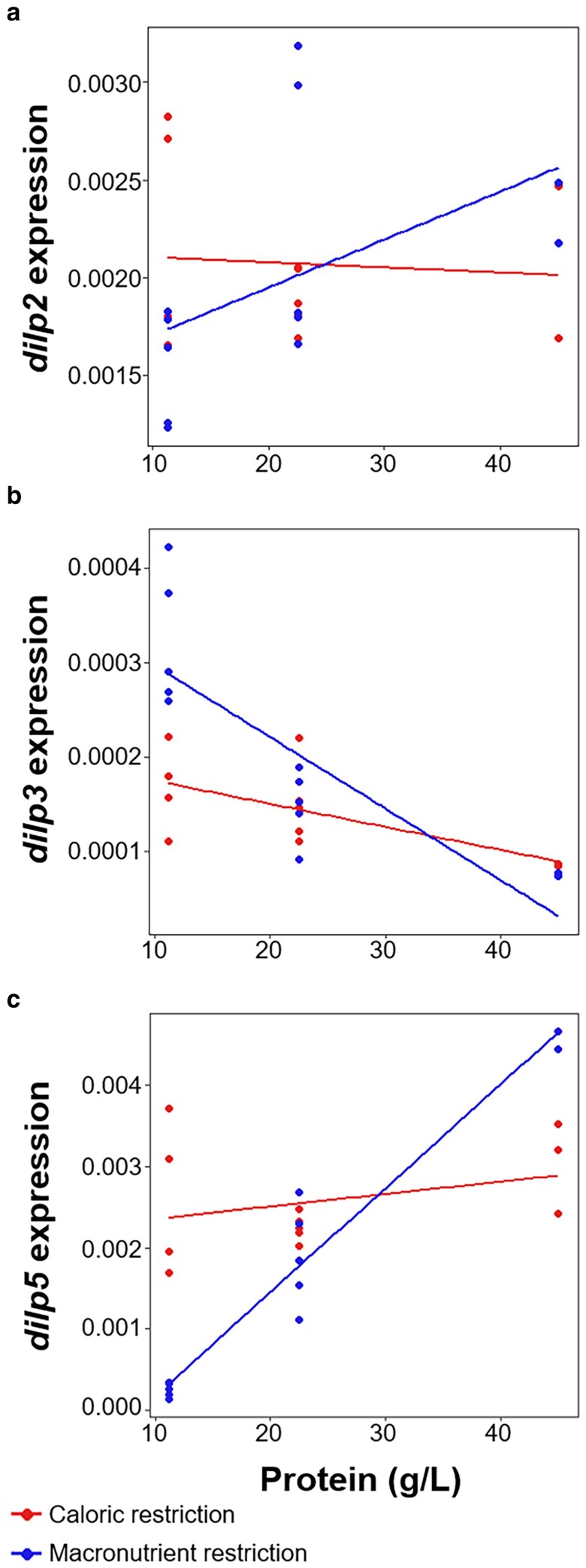
*Dilp* expression patterns across diets. Diluting the protein content of the larval diet via caloric restriction (red line) or macronutrient restriction (blue line) alters the expression patterns of *dilp2* (a)*, dilp3* (b), and *dilp5* (c) differently. Expression levels were normalized to an internal control, *Rp49. n* = 3–5 biological replicates of 15 larvae for all diets.

As protein concentration increased, *dilp2* expression increased but only on the macronutrient restriction diet (Fig. 2a, Table 3). On the caloric restriction diet, we did not find a significant relationship between protein concentration and *dilp2* expression, suggesting that *dilp2* expression is repressed by high levels of carbohydrates but may not be affected by protein concentration. For *dilp3*, expression decreased with increasing protein concentration on both the caloric restriction and macronutrient restriction diets, with a steeper decrease in expression observed when protein was diluted via macronutrient restriction (Fig. 2b, Table 3). This suggests that *dilp3* expression is induced by high levels of carbohydrates and repressed by high levels of protein. Conversely, *dilp5* expression increased significantly with increasing protein concentration on both diet types, although this increase was much more pronounced on the macronutrient restriction diet compared to the caloric restriction diet (Fig. 2c, Table 3). This expression pattern suggests that *dilp5* expression is induced by high levels of protein and repressed by high levels of carbohydrates. Taken together, these differing expression patterns suggest that the dILPs may function differently in response to changes in protein and carbohydrate concentration.

**Table 3.** The effect of dietary manipulations on dilp expression levels.

	Df	*dilp2* expression	*dilp3* expression	*dilp5* expression
		χ^2^ value	χ^2^ value	χ^2^ value
Protein	1	1.8740	31.3344***	53.720***
Diet	1	0.0393	6.8208**	10.734**
Protein:diet	1	4.4246*	9.2656**	38.587***

Significance codes: *P < 0.05, **P < 0.01, ***P < 0.001.

$R_{c}^{2}$ = 0.371, $R_{m}^{2}$ = 0.184.

### *dilp* mutants show a developmental delay that is dependent on both dietary protein concentration and diet type

**Fig. 3.**
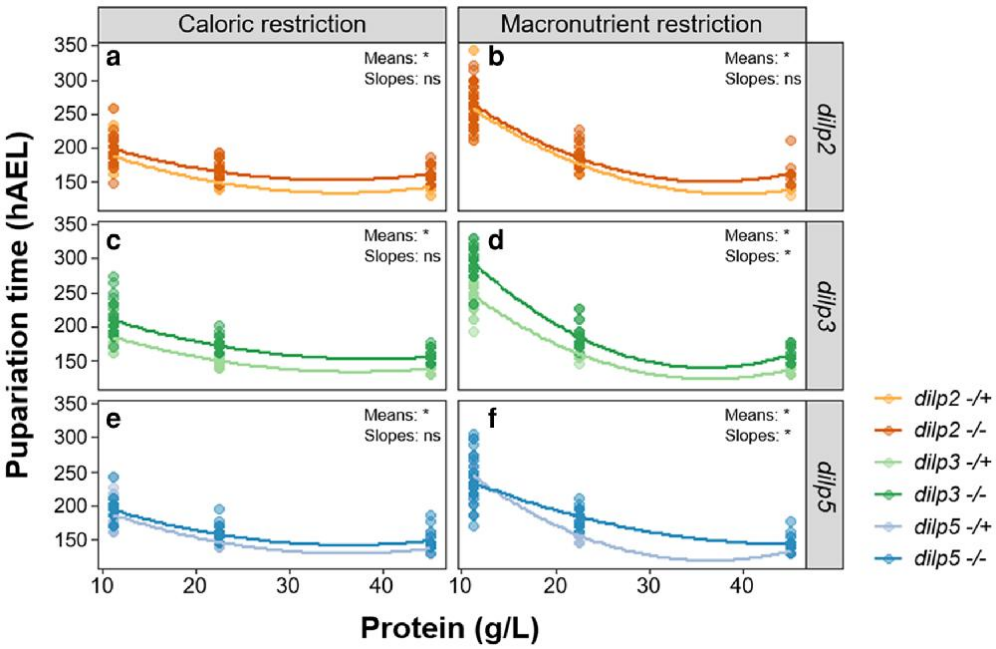
The developmental delay of *dilp* mutants is dependent on both dietary protein concentration and diet type. *dilp* mutants (−/−, darker lines) exhibit significantly longer mean pupariation times (means) compared to heterozygous controls (−/+, lighter lines) on both diet types (a to f). For all genotypes, pupariation time decreases with increasing protein concentration (a to f). The relationship between protein concentration and pupariation time (slopes) differs significantly between *dilp3* (d) and *dilp5* (f) mutants and their respective controls on the macronutrient restriction diet only; no significant differences in slopes were observed on the caloric restriction diet (a, c, e). hAEL = hours after egg lay. Asterisks (*) indicate a significant difference (*P* < 0.05) in the mean or slope of pupariation time between mutants and controls; ns = not significant. *n* = 5 for each diet across all genotypes and ≥13 individuals were tested for each treatment. Alpha blending was applied to indicate overlapping data points; darker areas represent higher point density.

**Fig. 4.**
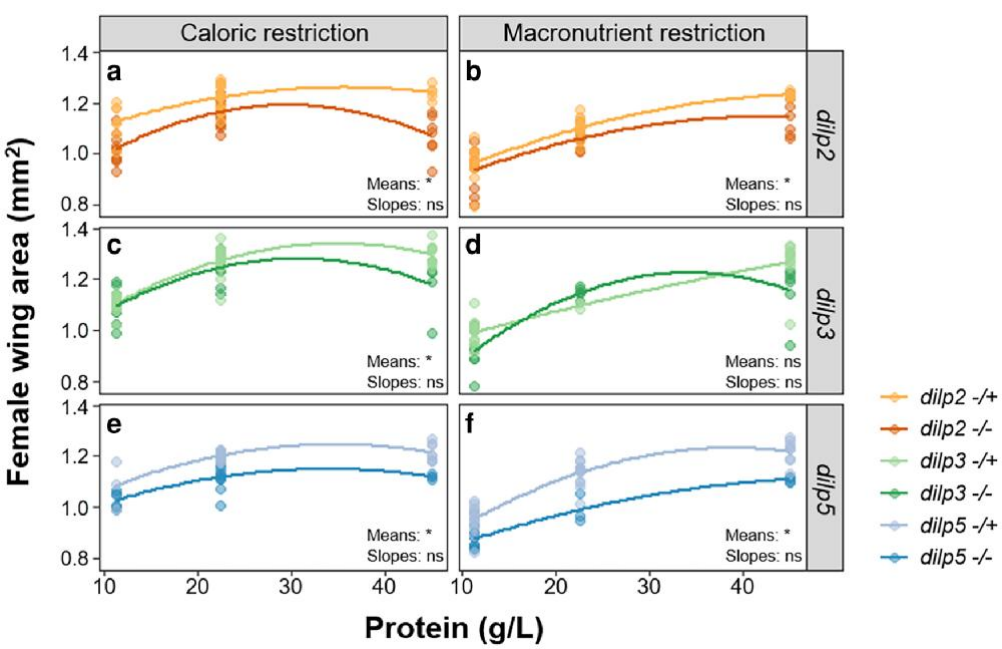
The relationship between dietary protein concentration and body size is not altered in *dilp* mutants, regardless of diet type. *dilp* mutants (−/−, darker lines) are significantly smaller than heterozygous controls (−/+, lighter lines) across both diet types (means), except for *dilp3* mutants on the macronutrient restriction diet (d), where no significant difference in size is observed. For all genotypes, wing area increases with increasing protein concentration (a–f). No significant differences in the relationship between protein concentration and wing area (slopes) were observed for any genotype on either diet type (a–f). Asterisks (*) indicate a significant difference (*P* < 0.05) in the mean or slope of wing area between mutants and controls; ns = not significant. *n* = 5 for each diet across all genotypes and ≥7 individuals were tested for each treatment. Alpha blending was applied to indicate overlapping data points; darker areas represent higher point density.

Given the striking differences we observed in the expression pattern of each *dilp*, both across protein concentrations and between diet types, we next investigated how individual null mutations in *dilps 2*, *3*, and *5* (Gronke et al. 2010) altered the development time and body size of larvae raised on either the caloric or macronutrient restriction diets. As observed for *w^1118^* (Fig. 1b, Table 2), the development time of all genotypes tested significantly decreased with increasing protein concentration on both diet types (Fig. 3, Table 4), and a more severe developmental delay was observed when larvae were raised on the low-protein macronutrient diets (Fig. 3b, d, and f, Table 4) compared to the low-protein caloric-restriction diets (Fig. 3a, c, and e, Table 4). Final body size, as measured by female wing area, significantly increased with increasing protein concentration for all genotypes on both the caloric and macronutrient restriction diets (Fig. 4, Table 5), as seen for *w^1118^* (Fig. 1d, Table 2).

**Table 4.** The effect of dietary manipulations on pupariation time in dilp mutants and heterozygotes.

	Df	*dilp2* pupariation time	*dilp3* pupariation time	*dilp5* pupariation time
		χ^2^ value	χ^2^ value	χ^2^ value
Protein	1	465.7739***	640.0862***	566.3273***
Diet	1	239.3838***	236.6184***	148.0803***
Mutation	1	20.6280***	104.0495***	12.3984***
Protein:diet	1	116.0699***	118.8703***	96.2492***
Protein:mutation	1	1.4665	5.2232*	7.5471**
Diet:mutation	1	1.7452	1.8269	0.4336
Protein:diet:mutation	1	0.0151	1.1902	2.1044

Significance codes: *P < 0.05, **P < 0.01, ***P < 0.001.

$R_{c}^{2}$ = 0.869, $R_{m}^{2}$ = 0.866.

**Table 5.** The effect of dietary manipulations on female wing area in dilp mutants and heterozygotes.

	Df	*dilp2* wing area	*dilp3* wing area	*dilp5* wing area
		χ^2^ value	χ^2^ value	χ^2^ value
Protein	1	68.5664***	98.3874***	145.0874***
Diet	1	51.6745***	60.5688***	83.0070***
Mutation	1	37.0625***	7.7068**	76.7701***
Protein:diet	1	21.9174***	9.3612**	29.1894***
Protein:mutation	1	3.3742	3.9279*	0.1857
Diet:mutation	1	3.2751	0.0024	1.6575
Protein:diet:mutation	1	0.0075	0.2500	0.6039

Significance codes: *P < 0.05, **P < 0.01, ***P < 0.001.

$R_{c}^{2}$ = 0.812, $R_{m}^{2}$ = 0.800.

When comparing the mean effect of individual *dilp* mutations to their heterozygous controls across all diets, we found that on both the caloric restriction and macronutrient restriction diets, *dilp2*, *dilp3*, and *dilp5* mutants all had a significant developmental timing delay compared to their heterozygous controls (Fig. 3, Table 4). These delays did not always result in significant changes in body size. For example, averaging across all caloric restriction diets, *dilp2*, *dilp3*, and *dilp5* mutants all had a significantly smaller wing area compared to their heterozygous controls (Fig. 4, a, c, and e, Table 5). However, when larvae were raised on the macronutrient restriction diets, only *dilp2* (Fig. 4b, Table 5) and *dilp5* (Fig. 4f, Table 5) mutants were significantly smaller than their corresponding heterozygous control. These data suggest that each dILP responds slightly differently to changes in protein concentration, and that this response differs depending on whether dietary protein is altered through caloric or macronutrient restriction.

Next, we analyzed whether *dilp* mutant larvae responded differently to changes in protein concentration compared to heterozygous controls by comparing the slopes of the relationships between protein content in the diet and either development time or wing area. When larvae were raised on the caloric restriction diets, no significant differences in the relationship between development time and protein concentration were observed for any of the genotypes (Fig. 3a, c, and e). However, on the macronutrient restriction diets, we found that *dilp3* (Fig. 3d) and *dilp5* (Fig. 3f) mutants responded differently to increasing protein concentration compared to their corresponding heterozygous controls. Specifically, we found that *dilp3* mutants had a steeper slope compared to heterozygous controls (Fig. 3d), indicative of a greater change in development time across protein concentrations, whereas *dilp5* mutants displayed a shallower slope compared to heterozygous controls (Fig. 3f), meaning that development time is less affected by protein concentration in this genotype.

**Fig. 5.**
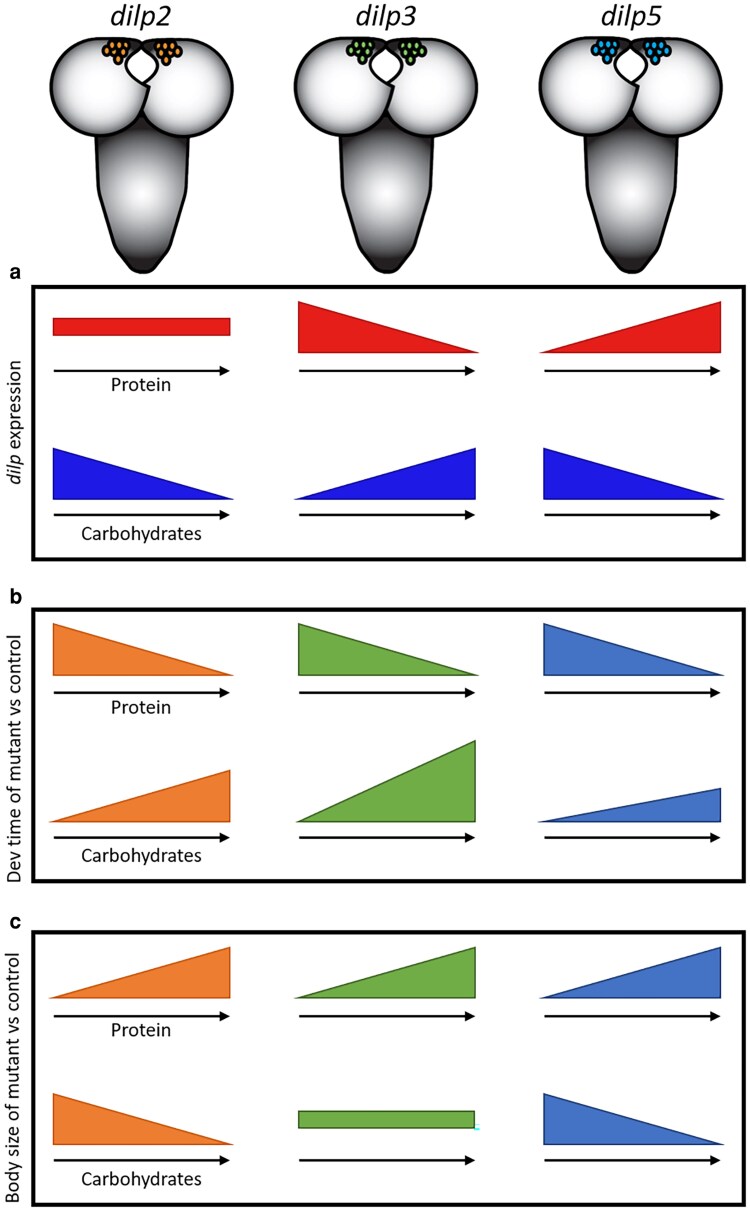
*dilps*  *2*, *3*, and *5* have different expression patterns and their mutations induce different phenotypic responses to changes in dietary protein and carbohydrates. *dilps 2*, *3*, and *5* differ in how their expression patterns (a) respond to changes in dietary protein concentration (under the caloric restriction diets) and carbohydrate concentration (under the macronutrient restriction diets). Similarly, null mutations in each of these 3 *dilps* produce different phenotypic responses for development time (b) and body size (c) phenotypes observed under caloric restriction diets or macronutrient restriction conditions. For all panels (a–c), the top row of graphs shows the changes in response to increasing protein concentration, and the bottom row of graphs shows the changes in response to increasing carbohydrate concentration. Note, for the *dilp* mutant phenotypes (b, c), the graphs show the change in phenotype compared to the corresponding heterozygous control.

For body size, no significant differences in the relationship between female wing area and protein concentration were observed between any of the genotypes on either the caloric or macronutrient restriction diets (Fig. 4). Thus, despite significant differences in the relationship between development time and protein concentration for some of the mutants analyzed, this did not lead to changes in the relationship between protein concentration and body size. Taken together, these results demonstrate the important but distinct roles of dILPs 2, 3, and 5 in responding to dietary protein concentration and adjusting development and growth accordingly (Fig. 5).

## Discussion

Approaches using nutritional geometry have significantly advanced our understanding of the role macronutrients play in animal fitness (Lee et al. 2008; Simpson and Raubenheimer 2012; Rodrigues et al. 2015; Alton et al. 2020). In *Drosophila*, nutritional geometry has revealed that both the caloric content and the ratio of protein to carbohydrates in the diet is important for regulating life history traits (Lee et al. 2008; Skorupa et al., 2008). These effects are thought to arise from differences in *dilp* expression profiles across the nutritional landscape; however, it is unclear if these differences are functionally significant (Kim and Neufeld 2015; Post and Tatar 2016; McDonald et al. 2021).

In this study, we diluted the protein content of the larval diet in two ways, caloric restriction and macronutrient restriction, and found striking differences in *dilp* expression patterns and development time both across protein concentrations and between diet types (for summary, see Fig. 5). Furthermore, our study revealed different growth phenotypes of individual *dilp* mutants raised on these diets. Taken together, our results support the hypothesis that changes in *dilp* expression are a key regulator of nutrient-dependent plasticity in *Drosophila* and suggest that each dILP may play slightly different roles in this process.

The observed expression patterns for *dilp3* and *dilp5* are consistent with previous studies which also found that *dilp3* expression declined with increasing protein concentration, whereas *dilp5* expression increased (Post and Tatar 2016; McDonald et al. 2021). However, we note some inconsistencies in the expression pattern of *dilp2* observed in our study compared to others (Post and Tatar 2016; McDonald et al. 2021). While we found that *dilp2* expression increased with increasing protein concentration on the macronutrient restriction diets, McDonald et al. (2021) have previously reported *dilp2* expression to decline with an increase in both protein and carbohydrates, which is also similar to the findings of Post and Tatar (2016). We did, however, find no significant change in *dilp2* expression across the caloric restriction diets, which has previously been shown (Ikeya et al. 2002; Geminard et al. 2009).

There are several possible explanations for these discrepancies. Firstly, our research measured *dilp* expression at the beginning of the third larval instar, whereas McDonald et al. (2021) took samples at the beginning of the wandering larval stage and Post and Tatar (2016) used adult flies in their study. As it is well known that IIS activity changes dynamically during development (Weger and Rittschof 2024), and different *dilps* are expressed at different life stages (Brogiolo et al. 2001; Ikeya et al. 2002; Semaniuk et al. 2020), it is difficult to directly compare results of studies that have collected samples at different life stages. Secondly, while previous studies have used at least 24 different diets covering a broad nutrient space with total food concentrations ranging from 45 g/L to 360 g/L and P:C ratios ranging from 0:1 to 1.9:1 (Post and Tatar 2016; McDonald et al. 2021), our study only included 5 different diets and thus covered a narrower nutrient space. The range of P:C ratios used in our study (1:10 to 1:2) fall within those previously used by Post and Tatar (2016) and McDonald et al (2021), whereas the total food concentrations of our diets (32 g/L to 128 g/L) goes below the lower limit of these previous studies, making direct comparisons between studies difficult. Future experiments should be designed such that *dilp* expression patterns can be measured in larvae raised across a broader range of diets to get a complete understanding of how macronutrients effect *dilp* expression levels. In addition, it would also be interesting to examine how dILP protein levels correlate to the observed mRNA levels.

Insulin/IGF-signaling is quite complex in *Drosophila*, with 7 insulin-like peptides (dILPs 1–7) that are all thought to act through a single receptor, InR (Brogiolo et al. 2001; Garofalo 2002). The extent of functional redundancy between dILPs, especially those with similar expression patterns, remains unclear. In a previous study, Gronke et al. (2010) showed that a combined *dilp2,3,5* gene-knockout had a more severe effect on development time, stress resistance, and lifespan than individual *dilp* mutants, a finding which supports the idea that these peptides are redundant in their activation of the IIS pathway. Our results suggest that there is some functional separation between dILPs. The striking differences in the expression patterns of *dilps 2*, *3,* and *5* that were observed in response to changes in protein concentration, together with the diverse phenotypes of individual *dilp* mutants raised on these diets, provides strong evidence that dILPs 2, 3, and 5 have distinct roles in regulating growth and development time in response to changes in dietary macronutrients.

Importantly, when one *dilp* is mutated the expression of the other IPC-expressed *dilps* is altered (Gronke et al. 2010). For example, in *dilp2* mutants, the expression of both *dilp3* and *dilp5* is upregulated (Gronke et al. 2010). This could explain why we did not observe a significant change in slope when *dilp2* mutants were raised on the macronutrient restriction diets. Conversely, in *dilp3* mutants both *dilp2* and *dilp5* are also downregulated (Gronke et al. 2010), which could explain why we observed a greater change in development time across protein concentrations in *dilp3* mutants. Future experiments should analyze the expression patterns of *dilps 2*, *3*, and *5* in individual mutants raised on both the caloric and macronutrient restriction diets to assess the extent to which the *dilps* compensate for one another.

One important question that remains is how do the dILPs, which are thought to act through a single receptor, mediate a range of phenotypes in response to changes in protein concentration? The unique expression patterns of *dilps 2*, *3*, and *5* that we observed in response to changes in protein concentration could explain the specific phenotypes observed. However, if the dILPs are all binding to the same receptor why does the phenotype change depending on which individual dILP is bound?

Recent evidence suggests that dILPs differentially activate InR to induce distinct cell signaling outputs that result in specific downstream phenotypes. In favor of this model, a previous study has shown that two insulin-like peptide family members in the mosquito *Aedes aegypti* exhibit partially overlapping biological activity and different binding affinities for the mosquito InR, despite both being expressed in the brain (Wen et al. 2010). Similarly, it has been shown in *Drosophila* S2 cells that dILP2 and dILP5 differentially stimulate cell signaling and glycogen phosphorylase (Post et al. 2018). Definitive proof comes from a recent study that demonstrates that dILP2 and dILP5 induce different conformational changes in InR (Cai et al. 2025). These differences in conformational changes result in dILP5 inducing higher levels of autophosphorylation in the Insulin Receptor than dILP2. Thus, individual dILPs are not strictly interchangeable.

Our study has provided important new insights into the distinct roles dILPs play in regulating nutrient-dependent plasticity in *Drosophila* that combines both expression data and important phenotypic information obtained from individual *dilp* mutants. Taken together, our results provide strong evidence to support the idea that the P:C ratio in the diet effects the expression of each *dilp* differently, and that these dILPs have differing, albeit overlapping, roles in the regulation of life history traits such as development time and body size. Future research should focus on a more in-depth analysis of both individual and combined *dilp* mutant animals on a wide nutritional landscape to tease apart the individual roles each dILP plays in regulating life history traits in response to changes in nutrition. Insights gained from this research will greatly enhance our understanding of the mechanisms controlling nutrient-dependent plasticity in *Drosophila.*

## Data Availability

All strains are available upon request. We affirm that all data necessary for confirming conclusions of this article are present within the article, figures, and tables. Datasets and R scripts are available at Figshare: https://doi.org/10.26180/29826530.
